# Reduced mRNA and Protein Expression Levels of Tet Methylcytosine Dioxygenase 3 in Endothelial Progenitor Cells of Patients of Type 2 Diabetes With Peripheral Artery Disease

**DOI:** 10.3389/fimmu.2018.02859

**Published:** 2018-12-06

**Authors:** Shi Zhao, Ting Jia, Yang Tang, Xiaotong Zhang, Hong Mao, Xiaojia Tian, Rui Li, Lu Ma, Guoxun Chen

**Affiliations:** ^1^Department of Endocrinology, Wuhan Central Hospital, Wuhan, China; ^2^School of Social Sciences, Nanyang Technology University, Singapore, Singapore; ^3^College of Health, Wuhan University, Wuhan, China; ^4^Department of Nutrition, University of Tennessee, Knoxville, TN, United States

**Keywords:** endothelial progenitor cells, tet methylcytosine dioxygenases, diabetes with peripheral artery disease, demethylation, diabetes

## Abstract

Endothelial progenitor cells (EPCs) with immunological properties repair microvasculature to prevent the complications in patients with diabetes. Epigenetic changes such as DNA methylation alter the functions of cells. Tet methylcytosine dioxygenases (TETs) are enzymes responsible for the demethylation of cytosine on genomic DNA in cells. We hypothesized that EPCs of diabetic patients with peripheral artery disease (D-PAD) might have altered expression levels of TETs. Subjects who were non-diabetic (ND, *n* = 22), with diabetes only (D, *n* = 29) and with D-PAD (*n* = 22) were recruited for the collection of EPCs, which were isolated and subjected to analysis. The mRNA and protein expression levels of *TET1, TET2*, and *TET3* were determined using real-time PCR and immunoblot, respectively. The *TET1* mRNA expression level in ND group was lower than that in the D and D-PAD groups. The *TET3* mRNA level in the ND group was higher than that in the D group, which was higher than that in the D-PAD group. The TET1 protein level in the D-PAD group, but not the D group, was higher than that in the ND group. The TET2 protein level in the D-PAD group, but not the D group, was lower than that in the ND group. The TET3 protein level in the ND group was higher than that in the D group, which was higher than that in the D-PAD group, which is the lowest among the three groups. The changes of TETs protein levels were due to the alterations of their transcripts. These probably lead to epigenetic changes, which may be responsible for the reductions of EPCs numbers and functions in patients with the D-PAD. The expression pattern of *TET3* mRNA and TET3 protein in EPCs may be a biomarker of angiopathy in diabetic patients.

## Introduction

The data collected in the Diabetes Control and Complications Trial initiated about three decades ago have demonstrated the importance of intensive diabetes treatment for the delay and slowdown of the progression of complications in patients with type 1 diabetes (1–3). The main mediator of complication has been consistently shown to be hyperglycemia (3). Circulating endothelial progenitor cells (EPCs) derived from the bone marrow play important roles in tissue repair to prevent or attenuate the development of diabetic complications (4, 5). The EPCs derived from the circulating mononuclear cells (MNCs) also have immunological properties such as presenting antigens and activating T cells (6, 7). Patients with diabetes mellitus have dysfunctions of EPCs, which may lead to reduced repair of their vascular system (8). The changes of EPC number and their functions have been attributed to macrovascular and microvascular complications, processes that may involve epigenetic mechanisms (8). Epigenetics defines the stable gene expression profile formed during development and cell proliferation, which is marked by the methylation profile of genomic DNA (9). Specific traits of epigenetics associated with type 2 diabetes have been identified and considered as potential biomarkers for the prevention and treatment of diabetes (10).

The DNA methylation generally occurs on the 5th carbon of cytosines of the dinucleotide CpG to form 5-methylcytosine (5mC), which happens in the cycle of demethylation and *de novo* methylation after the replication of the genome (9). The 5mC found at the cytosine of CpG dinucleotides has been considered to play a role in gene silencing as hypermethylation is associated with down-regulation of gene expressions (11). In mammalian cells, one pathway for the demethylation of cytosine to occur is mediated by the production of 5-hydroxymethylcytosine (5hmC), and followed by the base excision repair system to put the cytosine back into the position (11).

In humans with acute myeloid leukemia (AML), the *MLL* gene on 11q23 is fused to the LCX leukemia-associated protein with a CXXC domain gene on 10q22, which was cloned as Ten-eleven translocation methylcytosine dioxygenase 1 (TET1) (12). Later, through a computational search, it was found that TET1 was able to mediate the conversion of 5mC to 5hmC in an iron and α-ketoglutarate (2-oxoglutarate) dependent manner (13). The TET protein activity is also involved in the conversion of 5mC to 5-formylcytosine and 5-carboxylcytosine, two additional cytosine derivatives in genomic DNA (14). It has been thought that TET1 binds to CpG area to block the association of DNA methylation and convert 5mC to 5hmC during the embryonic stem cells differentiation and development (15). The 5hmC as an epigenetic modification reflects the pluripotent state of embryonic stem cells, and TET1 acts to determine their differentiation potential (16). So far, a family of three members (TET1, TET2, and TETE3) have been identified (16). Overexpression of TET2 in 293 cells leads to the increases of 5hmC level and DNA demethylation (17). Simultaneous deletion of both *Tet1* and *Tet3* results in a global loss of 5hmC and gain of 5mC in mouse embryos, indicating the roles of TET1 and TET3 in the formation of 5hmC (18). In addition, the deletion of *Tet1, Tet2, and Tet3* gene concurrently leads to the stop of stem cell reprograming (19).

It has been shown that both mRNA and protein levels of TET1, but not TET2 and TET3, are higher in the inferior parietal lobule of the psychotic patients than the control subjects (20). An elevation of 5hmC level has been observed at the promoter of glutamic acid decarboxylase 67 gene in the psychotic patients, a phenomenon that is associated with the reduction of the gene expression (20). In humans, the *TET2* expression level in skin wound tissue of diabetic patients is significantly higher than that in the wound of non-diabetic patients (21). This is associated with the elevation of α-ketoglutarate levels of the wound tissue, but not plasma and urine, in diabetic patients compared with those in the non-diabetic subjects (21). It has been thought that TET3 interacts with O-linked β-GlcNAc transferase to be exported out from the nuclei, which probably reduces its activity on chromatin (22). O-linked β-GlcNAc transferase has been thought to cause insulin resistance through the action on phosphoinositide production and modification of insulin receptor substrate in differentiated 3T3-L1 adipocytes (23). All these suggest that the 5hmC level could be altered in DNA of diabetic patients, and the expression levels of TET proteins may be associated these epigenetic changes.

Previously, we have shown that the amount of EPCs in patients with type 2 diabetes is negatively associated with the plasma HbA1c level (24). The functions of EPCs in type 2 diabetic patients with peripheral angiopathy are reduced (24). Others have observed the loss of 5mc in lens of patients with diabetic cataract, suggesting the involvement of TET proteins (25). This has been attributed to human lens epithelial damage through endoplasmic reticulum stress (25). The epigenome-wide analysis of a group of genes related to insulin resistance in visceral adipose tissue of morbidly obese insulin resistant and sensitive subjects have been compared to determine the methylation statuses (26). The promoter region of ZNF714 (Zinc Finger Protein 714) gene has been found to have lower methylation, which is associated with an increase of its transcription (26).

Here, we compared the mRNA and protein levels of *TET1, TET2*, and *TET3* in non-diabetic (ND) control subjects, patients with only type 2 diabetes only (D), and patients with diabetes peripheral artery disease (D-PAD). Results shown here indicated alterations of their mRNA and protein levels in EPCs of D-PAD subjects.

## Materials and Methods

### Reagents

Pure ethanol and isopropanol were obtained from Sinopharm (Beijing, China). Trizol, ultrapure Agarose, Superscript III RT kit, and SYBR quantitative real-time PCR (qPCR) mix were purchased from Invitrogen (ABI-Invitrogen/ThermoFisher, Carlsbad, CA). Anti-β-Actin (ab8227), anti-TET1 (ab 105475), anti-TET2 (ab94580), and anti-TET3 (ab139311) antibodies were obtained from Abcam (Cambridge, MA, USA). All other molecular biology and immunoblot reagents were obtained from MDL biotech Application Co (Beijing, China) unless described otherwise.

### Study Subjects

Patients of D (*n* = 29, 15 males and 14 females) and D-PAD (*n* = 22, 8 males and 14 females) groups were recruited during their visits in the clinic of the Endocrinology Department at the Central Hospital of Wuhan. ND subjects (*n* = 22, 9 males and 13 females) were recruited from the general population. Type 2 diabetes was determined using blood glucose cut-off values as defined by World Health Organization. PAD of the lower extremities was diagnosed on the basis of a history of claudication or rest pain, bilateral pulses examination and duplex ultrasound. Patients were graded according to the Leriche/Fontaine clinical classification of chronic lower extremity ischemia (27). Exclusion criteria for all subjects were any of the following clinical conditions: advanced microvascular diseases (diabetic retinopathy and nephropathy), auto-immune diseases, neoplasm, acute or chronic infections, recent (< 6 months) surgery or vascular intervention, age >80 years, recent (< 6 months) myocardial infarction, hemodialysis and use of immunosuppressive medication. All subjects were screened for cardiovascular risk factors to establish confounding effects, including: smoking, hypertension, BMI and dyslipidemia, and were measured ankle-brachial index (ABI). Plasma levels of glucose, glycated hemoglobin (HbA1c), triacylglycerol (TAG), and low density lipoprotein (LDL) in subjects after an over-night fasting were measured by standard clinical metabolic tests. Blood samples were collected during June 2016 to October 2016. The patients and control subjects were briefed about this study. All participants had signed the informed consent form before the study enrollment. The study protocol, #[2013]IEC(S031), was approved by the Institutional Review Board (IRB) in the Wuhan Central Hospital.

### EPCs Isolation and Characterization

**Figure 1:**
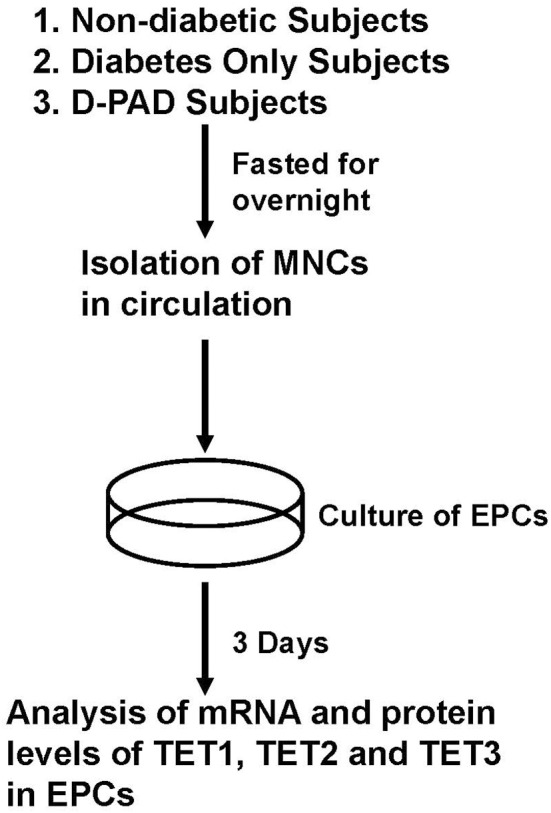
Procedures of isolation, preparation and analysis of EPCs from subjects in ND, D, and D-PAD groups. The recruited subjects were fasted overnight before their peripheral blood samples were collected for the collection of circulating mononuclear cells (MNCs). MNCs were cultured for 3 days as described in the Materials and Methods section. Total cell RNA and proteins were extracted and subjected to analysis of real-time PCR and immunoblot, respectively. ND, non-diabetic; D, Diabetes only; D-PAD, diabetes peripheral artery disease.

Figure 1 shows the procedure of sample collection and process. About 30 ml of peripheral blood samples were collected from ND, D, and D-PAD subjects by venipuncture and into EDTA vacutainers. The circulating MNCs were first isolated using Ficoll (catalog number: TBD2011H, Hao Yang Biological Manufacture Co. LTD, Tianjin, China) density-gradient centrifugation from human peripheral blood buffy coats as shown in Rehman et al. (28). Immediately after isolation, 1 × 10^6^ MNCs/ml of medium was plated on culture dishes coated with human fibronectin and maintained in endothelial basal medium (catalog number: CC-3156, Lonza, Basel, Switzerland) supplemented with EGM SingleQuots (catalog number: CC-417, Lonza) and 20% FCS (Catalog number: 16000-044, Gibco/Thermo Fisher Scientific). After being cultured for 3 days, non-adherent cells were removed by thorough washing with PBS. To confirm the EPC phenotype, adherent cells were incubated with 2.4 g/ml DiI-conjugated acetylated low-density lipoproteins (Dil-Ac-LDL, Invitrogen) at 37°C for 1 h and fixed with 4% paraformaldehyde for 10 min. After fixation, cells were incubated with FITC-labeled *Ulex europaeus* agglutinin I (catalog number: AL-1063, Vector, Olean, NY) for 1 h. Labeled cells were visualized with an inverted fluorescent microscope, and the adhered EPCs were determined by positive staining of both FITC–ulex-lectin and DiI-acLDL. Cell nuclei were stained with DAPI. The fluorescent results indicated that more than 95% of adherent cells were acLDL(+)ulex-lectin(+), demonstrating the high purity of EPCs used in the studies.

### RNA Isolation

For RNA isolation, each cell sample was dissolved in 1 ml Trizol reagent. After that, 0.5 ml of chloroform was added to the mixture, which was vortexed and then, placed on ice for 5 min for settlement. The mixture was centrifuged at 12,000 × g and 4°C for 15 min. The RNA containing upper aqueous phase was carefully removed and mixed with 0.5 ml of isopropanol at room temperature for 10 min. For RNA precipitation, the mixture was centrifuged at 12,000 × g and 4°C for 10 min. The RNA pellet was washed once with 1 ml 75% Ethanol and centrifuged at 7,500 × g and 4°C for 5 min to precipitate it. The pellet was air-dried and dissolved in 50 μl DEPC-treated H_2_O until further process.

### cDNA Synthesis

For the cDNA synthesis, the following components were included in a 20-μl reaction, 200 ng RNA in 10 μl, 1 μl of 50 μM oligo-dT, 1 μl of 50 μM random hexamer, 1 ul 10μM dNTP mix, 4 μl 5X first-strand buffer (250 mM Tris-HCl, pH 8.3, 375 mM KCl, 15 mM MgCl_2_), 2 μl 100 mM DTT, and 1 μl Superscript III reverse transcriptase (200 U). The RNA, oligo-dT and random hexamer were mixed first, incubated at 65°C for 5 min, and immediately put on ice. After that, 5 × first-strand buffer, DTT and reverse transcriptase were added, mixed well by pipetting gently up and down, and then, the mixture was incubated at 42°C for 60 min. This reverse transcription reaction was then incubated at 85°C for 10 min to inactivate the reverse transcriptase. The synthesized cDNA was stored at −20°C before being used for qPCR analysis.

### Quantitative qPCR Analysis

Each SYBR green based qPCR reaction contains, in a final volume of 20 μl, 2 μl of cDNA from 20 ng of reverse transcribed total RNA, 2 μl mixture of the forward and reverse primers at 5 μM total concentration, 10 μl of 2 × SYBR Green PCR Master Mix (Applied Biosystems) and 6 μl H_2_O. The sequences of primers used for the detection of the indicated genes are shown in Table 1. Triplicate PCR reactions were carried out using 7900 Real-Time PCR System (Applied Biosystems). The reaction conditions were 95°C for 2 min, followed by 40 cycles of 94°C for 20 s (s), 65°C for 20 s and 65°C for 30 s. The gene expression level was normalized to that of invariable control gene, β-actin. Data were presented as the minus cycle threshold (ΔCT, the Ct of gene of interest minus the Ct of β-actin) (29, 30).

**Table 1:** Sequences of primers (5′ to 3′) used in the real-time PCR for the detection of *TET1, TET2, TET3* and the control gene *ACTB* (β-actin gene).

**Genes**	**Forward primers (5^′^ to 3^′^)**	**Reverse primers (5^′^ to 3^′^)**	**Gene bank**
*TET1*	CATCAGTCAAGACTTTAAGCCCT	CGGGTGGTTTAGGTTCTGTTT	NM_030625
*TET2*	GATAGAACCAACCATGTTGAGGG	TGGAGCTTTGTAGCCAGAGGT	NM_001127208
*TET3*	TACCAACCGCCGCACGCAC	AGCCGCTCCTTGTCCCCAC	NM_001287491
*ACTB*	GACAGGATGCAGAAGGAGATTACT	TGATCCACATCTGCTGGAAGGT	NM_001101.4

### Western Blot Analysis

To lyse the cells, 0.1 ml of RIPA buffer was used to suspend every 1 × 10^6^ EPCs, and the mixture was kept on ice for 20 min. The lysates were centrifuged at 12,000 × g and 4°C for 10 min. The supernatant containing EPC total cellular proteins was stored at −80°C until being used. Total cellular proteins (50 μg) of the EPCs were separated in 10% sodium dodecyl sulfate polyacrylamide gel at 8 v/cm. The proteins in the gels were transferred to PVDF membrane (Millipore, Norcross, GA, USA). The PVDF membranes were incubated in TBST containing 5% non-fat milk for 1 h at room temperature, and then incubated in 5% dry-milk TBST containing 1 μg/ml of anti-TET1, anti-TET2, or anti-TET3 antibody at 4°C for overnight. After that, membranes were washed three times in TBST for 5 min each, and then, incubated in TBST containing 8% non-fat milk with goat anti-rabbit IgG conjugated with horseradish peroxidase (1/5,000 dilution) for 1 h at room temperature. After that, membranes were washed three times in TBST for 5 min each, and then, the bound-secondary antibody was detected using chemiluminescence (ECL Western Blotting Substrate, Thermo Scientific) through exposure to X-ray films (Phenix Research Products, Candler, NC). The films were scanned at 300 dpi using a HP ScanJet 2200 Scanner. The densities of the corresponding protein bands were normalized to that of the β-actin of the same sample using ImageJ software (National Institute of Health, MD).

### Statistical Analysis

Data of both male and females were analyzed together. Multiple linear regression analyses was conducted to determine the differences among three groups and between two groups (D and D-PAD groups) on the expression of mRNA and protein levels of *TET1, TET2*, and *TET3* genes after being adjusted for sex, BMI, ABI, HbA1c, TAG, and LDL. One-way ANOVA with LSD *post-hoc* statistical analysis was performed when more than two groups were compared. All statistical analyses were performed using SPSS statistical software (IBM, version 17). Data were presented as means ± S.E.M. A *p* < 0.05 was considered statistically significant.

## Results

### Measurements of Anthropometric and Plasma Parameters of Subjects in ND, D, and D-PAD Groups

Table 2 shows the baseline anthropometric and plasma parameter data of subjects in ND, D, and D-PAD groups. The data of male and female subjects were analyzed together. The conclusions did not differ when they were analyzed separately. Patients in D-PAD group had similar age, BMI, and levels of HbA1c, TG, LDL, and glucose as those in D group. The subjects in ND group had lower plasma levels of HbA1c and glucose, and were younger than those in D-PAD and D groups. Patients in D-PAD group had higher ABI value than subjects in the ND group.

**Table 2:** Baseline anthropometric and plasma parameters of subjects in D-PAD, D, or ND groups collected after fasting for overnight.

**Groups (Number)**	**ND (22, 9M/13F)**	**D (29, 15M/14F)**	**D-PAD (22, 8M/ 14F)**
Age	39 ± 3.2	62 ± 2.0 ^*^ (*P* < 3.7E-08)	66 ± 1.9 ^*^ (*P* < 3.8E-09)
BMI	22.3 ± 0.6	23.3 ± 0.4	24.3 ± 0.6 ^*^ (*P* < 0.032)
HbA1c (%)	5.6 ± 0.1	8.1 ± 0.3 ^*^ (*P* < 6.7E-09)	8.3 ± 0.4 ^*^ (*P* < 8.8E-07)
ABI	1.168 ± 0.026	1.108 ± 0.025	1.037 ± 0.032 ^*^ (*P* < 0.0030)
TAG (mmol/L)	1.6 ± 0.3	1.8 ± 0.3	1.6 ± 0.2
LDL(mmol/L)	2.5 ± 0.2	2.7 ± 0.2	2.5 ± 0.2
Glucose (mmol/L)	5.0 ± 0.2	9.3 ± 0.9 ^*^ (*P* < 0.0002)	9.6 ± 0.7 ^*^ (*P* < 1.3E-06)

*Data were presented as mean ± SEM*.

* *P < 0.05 for comparing the indicated group with the control group. The numbers of subjects in each group were included in the parenthesis. BMI, body mass index; ABI, ankle-brachial index; D, Diabetes only; D-PAD, diabetes peripheral artery disease; F, female; HbA1c, glycated hemoglobin A1C; LDL, low density lipoprotein; M, male; ND, non-diabetic; TAG, triacylglycerol*.

### Comparison of Expression Levels of *TET1, TET2*, and *TET3* mRNA in EPCs of Subjects in ND, D, and D-PAD Groups

**Figure 2:**
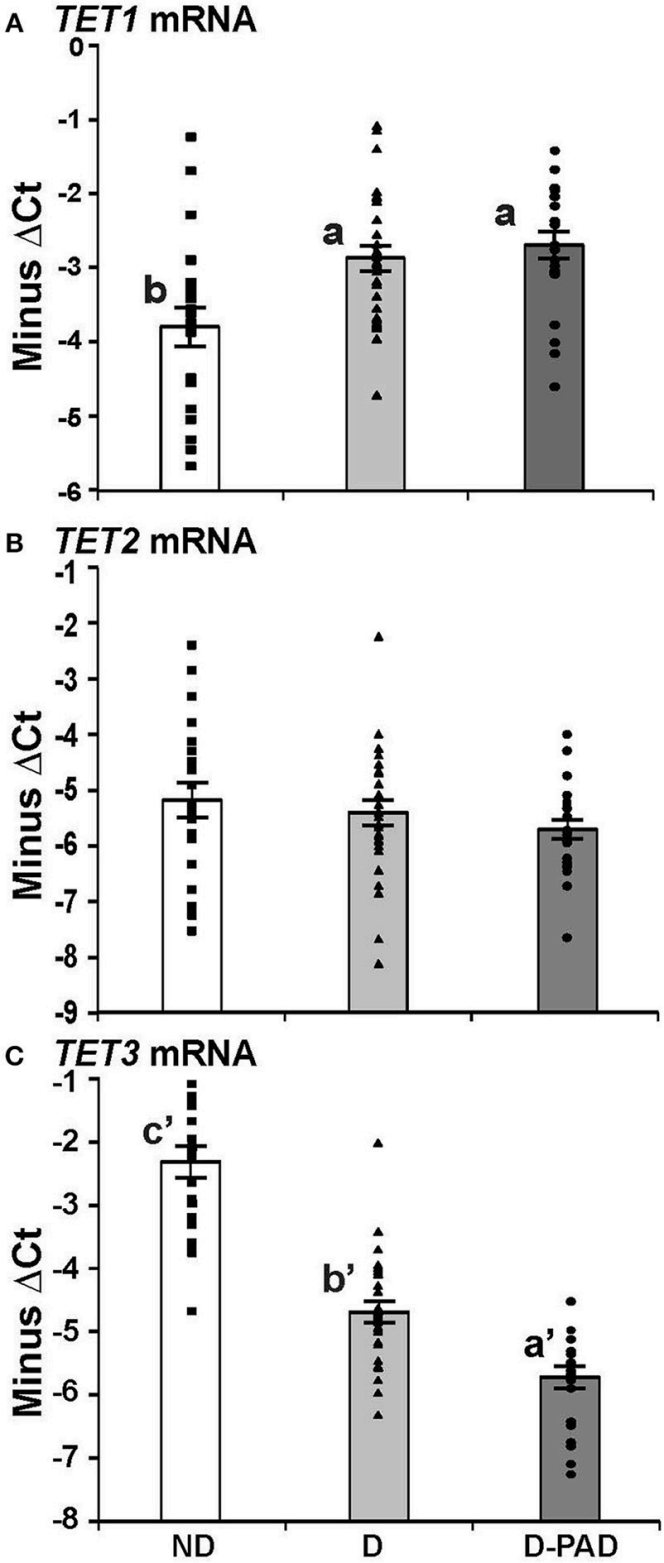
Comparison of the relative expression levels of TET1 **(A)**, TET2 **(B)**, and TET3 **(C)** mRNA in EPCs of subjects in ND, D and D-PAD groups. EPCs were isolated from subjects in ND (*n* = 22), D (*n* = 28), and D-PAD (*n* = 22) groups and cultured as described in the Material and Methods section. Total RNA of EPCs was isolated and subjected to real-time PCR analysis. The results were presented as mean ± SEM of the –ΔCT (the CT of β-actin—the CT of indicated transcript). The distribution ranges of the –ΔCT values of their genes were shown with the columns in the graph. The data were analyzed with one way ANOVA (a > b, *P* < 0.0012 for comparing ND and D-PAD groups, and *P* < 0.0034 for comparing ND and D groups; and a′ < b′ < c′, *P* < 7.1E-11 for comparing ND and D groups, and *P* < 7.2E-05 for comparing D and D-PAD groups). ND, non-diabetic; D, Diabetes only; D-PAD, diabetes peripheral artery disease.

Figure 2 shows the relative mRNA levels of *TET1* (A), *TET2* (B), and *TET3* (C) genes presented as –ΔCt (Ct of β-actin—Ct of the indicated gene) in the forms of column and scatter plot. As shown in Figure 2A, the relative *TET1* mRNA expression level in EPCs of the subjects in ND group was lower than those in D and D-PAD groups (a > b). The relative *TET1* mRNA levels of those subjects in the D and D-PAD groups were not different from each other. As shown in Figure 2B, there was a reduction trend of relative *TET2* mRNA expression level in EPCs of the D-PAD group in comparison with that of the ND group. However, it did not reach statistical significance. Therefore, the *TET2* mRNA levels in EPCs of subjects of in three groups were not different from each other.

On the other hand, the relative *TET3* mRNA levels in EPCs of subjects in ND group were higher than that of the D group as shown in Figure 2C. More importantly, the relative *TET3* mRNA levels in EPCs of subjects in the D group were higher than that in the D-PAD group. The relative *TET3* mRNA level in D-PAD group was the lowest among the three groups (a′ < b′ < c′).

### Comparison of Relative *TET1, TET2*, and *TET3* mRNA Levels in Three Groups (ND, D, and D-PAD Group) *via* Multiple Linear Regression Analysis Adjusted for Anthropometric and Plasma Parameters

The results of multiple linear regression analysis were shown in Table 3. The differences of the relative expression of *TET1* mRNA, and *TET3* mRNA between the ND group and the D or D-PAD groups were statistically significant. Compared with that in the ND group, the average relative *TET1* mRNA values of D and D-PAD groups increased by 1.212 and 1.407, respectively, after being adjusted for sex, BMI, ABI, HbA1c, TAG, and LDL. Compared with that in the ND group, the average relative *TET3* mRNA values in the D and D-PAD groups decreased by 2.161 and 3.158, respectively, after being adjusted for sex, BMI, ABI, HbA1c, TAG, and LDL.

**Table 3:** Multiple linear regression analysis to compare the *TET1* and *TET3* mRNA levels in ND group with that in D and D-PAD groups.

**Dependent variable**	**Independent variable**	***beta***	***Std. error***	***t***	***P***
***TET1*** **mRNA**					
	D	1.212	0.423	3.019	0.004
	D-PAD	1.407	0.466	2.863	0.006
***TET3*** **mRNA**					
	D	−2.161	0.408	−5.296	< 0.001
	D-PAD	−3.158	0.449	−7.031	< 0.001

*ND, non-diabetic; D, Diabetes only; D-PAD, diabetes peripheral artery disease*.

### Comparison of Levels of TET1, TET2, and TET3 Proteins in EPCs of Subjects in ND-D and D-PAD Groups

**Figure 3:**
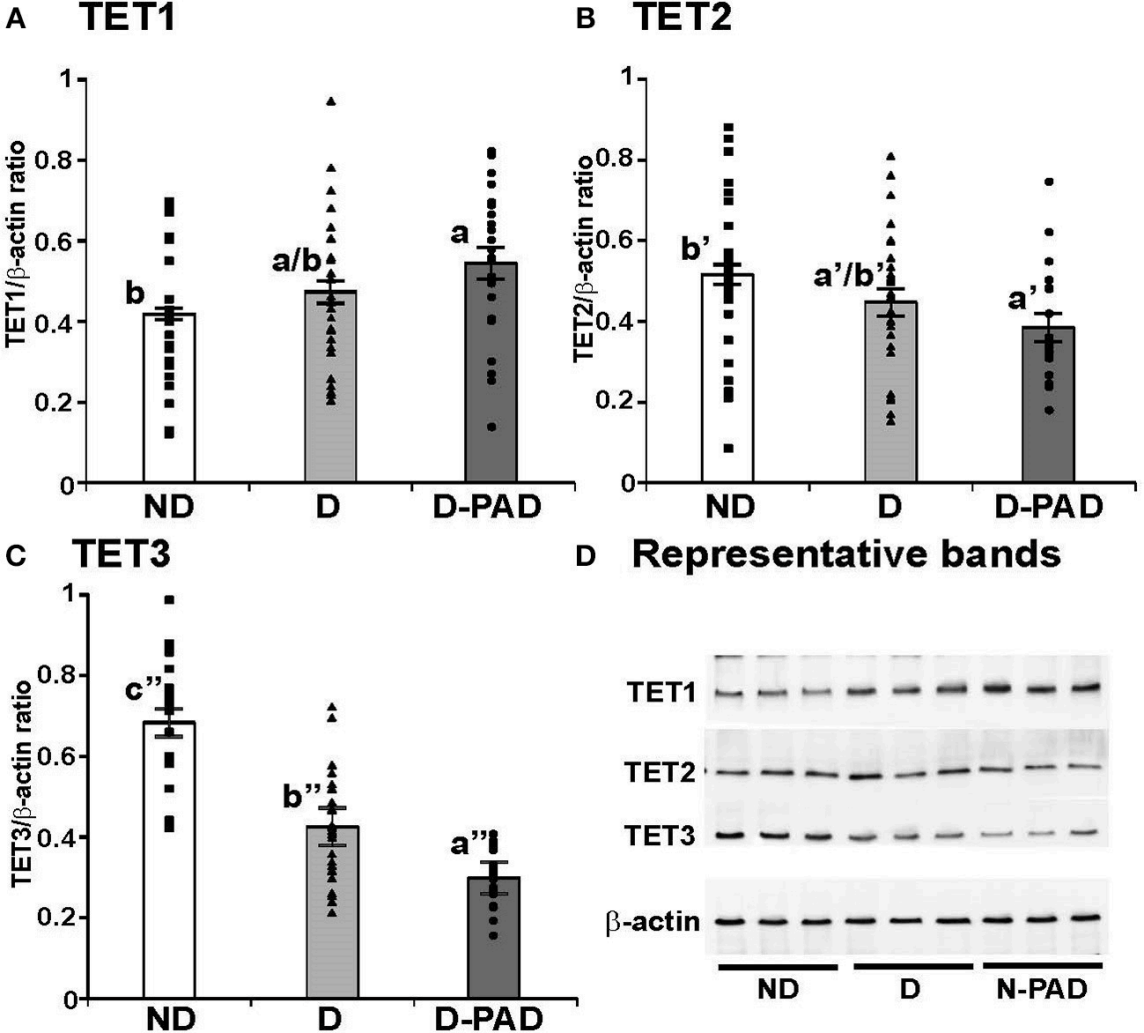
Comparison of the relative expression levels of TET1 **(A)**, TET2 **(B)**, and TET3 **(C)** protein, and representative immunoblot results of these three proteins **(D)** in EPCs of subjects in ND, D and D-PAD groups. EPCs were isolated from subjects in ND (*n* = 22), D (*n* = 28), and D-PAD (*n* = 22) groups and cultured as described in the Material and Methods section. Total cellular proteins of EPCs were isolated and subjected to immunoblot using the specific antibodies to β-actin (the invariable loading control), TET1, TET2, and TET3. The distribution ranges of the ratios of the densities of the indicated protein to β-actin were shown with the columns in the graph. The results were presented as mean ± SEM of the ratios of the indicated protein to β-actin. **(D)** shows the representative immunoblot images of TET1, TET2, TET3, and the house-keeping protein β-actin using three protein samples each in ND, D, and D-PAD groups. The data were analyzed with one way ANOVA (a > b, *P* < 0.03 for comparing ND and D-PAD groups; a′ < b′, *P* < 0.02 for comparing ND and D-PAD groups; a″ < b″ < c″, *P* < 1.12E-07 for comparing ND and D groups, and *P* < 0.0002 for comparing D and D-PAD groups). ND, non-diabetic; D, Diabetes only; D-PAD, diabetes peripheral artery disease.

To determine whether the changes of *TET* mRNA transcripts correspond to the alterations of their protein amounts, the expression levels of TET1, TET2, and TET3 proteins in these three groups were determined using immunoblot, normalized to that of the house-keeping gene, β-actin, and presented as the ratio of the density of indicated protein to that of β-actin as shown in Figure 3. Figure 3A shows the relative expression levels of TET1 protein in EPCs of the three groups. The relative TET1 protein level in EPCs of the subjects in the D-PAD group, but not that of the D group, was higher than that in the ND group (a > b). This result matched partially with the mRNA data (Figure 2A).

Interestingly, the relative TET2 protein level in EPCs of the patients in the D-PAD group, but not that of the D group, was lower than that of the ND group as shown in Figure 3B (a′ < b′). This result matched the reduction trend of the *TET2* mRNA shown in Figure 2B.

Figure 3C shows that the relative expression level of TET3 protein in EPCs of the subjects in ND group was higher than that of the patients in the D group, which was even higher than that of the patients in the D-PAD group (a″ < b″ < c″). TET3 protein level in the D-PAD group was the lowest in the three groups.

Figure 3D shows the representative immunoblot images of TET1, TET2, TET3, and the house-keeping protein β-actin using three EPC protein samples each in ND, D, and D-PAD groups. The results supported the data shown in Figures 3A–C.

### Comparison of TET1, TET2, and TET3 Protein Levels in Three Groups (ND, D, and D-PAD) *via* Multiple Linear Regression Analysis Adjusted for Anthropometric and Plasma Parameters

Table 4 showed the results of the multiple linear regression to compare the relative expression levels of TET proteins in the ND groups with that in the D and D-PAD groups. The differences of the relative expression levels of TET1, TET2, and TET3 proteins in the three groups were statistically significant. Compared with that of the subjects in the ND group, the average relative value of TET1 protein in the D-PAD group increased by 0.23 after being adjusted for sex, BMI, ABI, HbA1c, TAG, and LDL. Compared with that of the subjects in the ND group, the average relative value of the TET2 protein level in the D-PAD group decreased by 0.196 after being adjusted for sex, BMI, ABI, HbA1c, TAG, and LDL. Compared with that of the subjects in the ND group, the average relative values of TET3 protein levels in the D and D-PAD groups decreased by 0.261 and 0.37, respectively, after being adjusted for sex, BMI, ABI, HbA1c, TAG, and LDL.

**Table 4:** Multiple linear regression analysis to compare the TET1, TET2, and TET3 protein levels in ND group with that in D and D-PAD groups.

**Dependent variable**	**Independent variable**	***beta***	***Std. error***	***t***	***P***
**TET1 PROTEIN**					
	D-PAD	0.230	0.083	2.763	0.008
**TET2 PROTEIN**					
	D-PAD	−0.196	0.087	−2.268	0.027
**TET3 PROTEIN**					
	D	−0.261	0.055	−4.715	< 0.001
	D-PAD	−0.370	0.061	−6.080	< 0.001

*ND, non-diabetic; D, Diabetes only; D-PAD, diabetes peripheral artery disease*.

### Comparison of *TET* mRNA and TET Protein Levels in the D and D-PAD Groups *Via* Multiple Linear Regression Analysis After Adjusted for Anthropometric and Plasma Parameters

Table 5 shows the results of multiple linear regression analysis to compare relative *TET* mRNA and protein levels in the D with that in the D-PAD groups. The differences of relative *TET3* mRNA and protein levels between D and D-PAD groups were statistically significant. Compared with the values in the D group, the average relative values of *TET3* mRNA and TET protein expression levels in the D-PAD group decreased by 1.137 and 0.125, respectively, after being adjusted for sex, BMI, ABI, HbA1c, TAG, and LDL. Compared with that in the D group, the average relative value of the *TET3* mRNA level in the D-PAD group decreased by 1.939 for each unit increase of ABI after adjusted for sex, BMI, HbA1c, TAG, and LDL.

**Table 5:** Multiple linear regression analysis to compare the *TET3* mRNA and TET3 protein levels in D group with that in D-PAD group.

**Dependent variable**	**Independent variable**	***beta***	***Std. error***	***t***	***P***
***TET3*** **mRNA**					
	ABI	−1.939	0.934	−2.075	0.044
	D-PAD	−1.137	0.255	−4.457	< 0.001
**TET3 PROTEIN**					
	D-PAD	−0.125	0.035	−3.579	0.001

*ND, non-diabetic; D, Diabetes only; D-PAD, diabetes peripheral artery disease*.

## Discussion

Here, the expression levels of *TET1, TET2*, and *TET3* mRNA and their proteins in EPCs of ND, D and D-PAD subjects were compared. Our results demonstrated that *TET1* and *TET3* mRNA and proteins in EPCs of these three groups were expressed differently. It indicates that the epigenetic changes might have occurred during the development and progression of type 2 diabetes, which may be responsible for the reductions of EPCs number and their functions in patients of D-PAD groups.

The expression patterns of TET1, TET2, and TET3 proteins in EPCs of the three groups appeared to match well with that of their mRNA in the same groups. This indicates that the changes of TETs protein levels were mainly due to the alterations of their mRNA expression. The *TET1* and *TET2* mRNA and their proteins levels in EPCs of the D and D-PAD groups were not significantly different from each other, indicating that their limited association with the development of PAD. On the other hand, the *TET3* mRNA and TET3 protein levels in EPCs of D-PAD patients were lower than that of the D group, which was lower than that of the control group, the group with the highest TET3 mRNA and protein levels. This result indicates that the expression levels of *TET3* mRNA and TET3 protein in EPCs decrease along with the progression of the diabetes mellitus. The correlation of the decrease of *TET3* mRNA expression and the increase of ABI values further supports this conclusion. The association of TET3 expression with the PAD development suggests the possibility of using this phenomenon as a biomarker for the D-DAP diagnosis.

The *TET1* mRNA levels in the D and D-PAD groups and its protein level in the D-PAD were higher than that in the ND group. The elevated TET1 protein level suggests that an increase of amount of DNA 5hmC in EPCs of the D-PAD group. This assumption is supported by a recent report showing that global DNA 5hmC level in the peripheral blood of uncontrolled diabetic patients is higher than that of well-controlled patients and healthy individuals, which correlates with the plasma level of HbA1c (31). If the uncontrolled diabetic patients are in the same category as our D and D-PAD group, the results of our study and their observation seem to support each other well. It suggests that the alteration of *TET1* mRNA and TET1 protein expression levels can be a biomarker for the development of type 2 diabetes.

However, in the same report, the *TET1* mRNA level in the peripheral blood of uncontrolled diabetic patients is higher than that of control subjects, which is higher than that of the well-control diabetic subjects (31). This does not support any association of diabetes or the DNA 5hmC amount with the expression level of *TET1* mRNA. In addition, the DNA 5mC level in the uncontrolled diabetic patients is also higher than that in the well-controlled diabetic and control subjects (31). This phenomenon seems to indicate that the increase of *TET1* mRNA expression can only be used to explain part of the picture, which led the authors to suggest that additional mechanisms such as DNA oxidation may contribute to the changes of DNA 5mC and 5hmC (31). The oxidative state may be related to the production of 5hmC levels as vitamin C has been shown to stimulate the formation of 5hmC in cultured mouse embryonic fibroblasts, probably through reducing the inactive Fe^3+^ to the active Fe^2+^ for the activity of TETs (32–34). Unfortunately, the cited study only measured *TET1* mRNA expression level, but not that of *TET2* and *TET3* in the peripheral blood (31). As shown in our study, the protein levels of TET3 in EPCs of the D and D-PAD groups were lower than that of the ND group. The reduction of TET3 protein expression level may increase the amounts of 5mC in those genomic places relying on TET3 for the production of 5hmC. Whether the DNA oxidation or change of relative proportions of TET1 and TET3 contributes to the alteration of global DNA methylation status remains to be determined in future work.

The existence of 5mC and 5hmC allows variations of DNA structure, which leads to more variables than cytosine alone (35). The alterations of TETs expression levels in the EPCs of the D-PAD group imply the contribution of epigenetic to the reduction of EPC number and cellular functions along with the development of diabetes and complications. The remaining questions are how these changes of TET expression levels lead to alterations of cellular functions; what the regulatory mechanisms of these TETs expression are in response to the development of disease stages; what the physiological consequences of the induction of TET1 and reduction of TET3 are. In addition, whether the increase of 5hmC globally in the whole genome or locally in particular genes remains to be further investigated. The variation of 5mC and 5hmC amounts in a gene's promoter locally may be critical for its regulation. Recently, it has been shown that TET2 can be phosphorylated by AMP activated protein kinase, a process that is impeded in the presence of high glucose through the reduction of TET2 protein stability (36). This shows the importance of post-translational modification in the regulation of activities of TET proteins. Obviously, more studies are needed to explore the potential uses of these findings for the prevention and treatment of diabetes and its complications. This will be helpful for those patients with diabetes.

Our findings here have shown a potential epigenetic mechanism for the development of complications in patients with diabetes. One limitation here is that the age of subjects in the ND group is younger than that of the patients in D and D-PAD groups. However, this does not affect the conclusion drawn from the *TET3* mRNA and proteins as its levels in EPCs of D-PAD patients are lower than that of D group, which patients in both groups have no difference in age. Another limitation is that data of male and female subjects were analyzed together. This was due to limited number of subjects in both sex. Nevertheless, additional studies using age controlled subjects and more subject numbers in both sex are warranted in the future.

As far as we know, our data, for the first time, demonstrated an association of the expression levels of TETs in EPCs with the development of PAD. It is urgent to determine whether the changes of TET3 expression is the consequence of PAD development or the cause of it. In addition, the epigenetic changes in cells of diabetic patients seemed to be present in other cells as shown in the introduction section. This appears to indicate that some general factors presenting in diabetic patients contribute to the epigenetic changes. These factors may cause the increase of TET1 and decrease of TET3 expression levels, which become possible intervention points for the prevention and treatment of diabetes and its complications. It will be interesting to find out whether the changes of TET1 and TET3 expression levels in the EPCs will be reversed in diabetic patients treated with bariatric surgery or delayed in those treated with drugs for the control of blood glucose. When specific tools such as specific inhibitors or activators of TETs are developed and available, they may be tested to prevent or intervene the onset or progress of complications associated with diabetes.

## Author Contributions

SZ and GC designed the projects and experiments. TJ and HM collected the samples, preparations, and performed the experiments. YT, XZ, XT, and LM analyzed the data. SZ, RL, and GC organized the results and wrote the paper. All authors reviewed and approved this manuscript.

### Conflict of Interest Statement

The authors declare that the research was conducted in the absence of any commercial or financial relationships that could be construed as a potential conflict of interest.
